# The Effect of Dissolved Polyunsaturated Aldehydes on Microzooplankton Growth Rates in the Chesapeake Bay and Atlantic Coastal Waters

**DOI:** 10.3390/md13052834

**Published:** 2015-05-06

**Authors:** Peter J. Lavrentyev, Gayantonia Franzè, James J. Pierson, Diane K. Stoecker

**Affiliations:** 1 Department of Biology, University of Akron, Akron, OH 44325, USA; E-Mail: franze@uakron.edu; 2 Horn Point Laboratory, University of Maryland Center for Environmental Science, Cambridge, MD 21613, USA; E-Mails: jpierson@hpl.umces.edu (J.J.P.); stoecker@umces.edu (D.K.S.)

**Keywords:** polyunsaturated aldehydes, microzooplankton, growth, ciliates, dinoflagellates

## Abstract

Allelopathy is wide spread among marine phytoplankton, including diatoms, which can produce cytotoxic secondary metabolites such as polyunsaturated aldehydes (PUA). Most studies on diatom-produced PUA have been dedicated to their inhibitory effects on reproduction and development of marine invertebrates. However, little information exists on their impact on key herbivores in the ocean, microzooplankton. This study examined the effects of dissolved 2*E*,4*E*-octadienal and 2*E*,4*E*-heptadienal on the growth rates of natural ciliate and dinoflagellate populations in the Chesapeake Bay and the coastal Atlantic waters. The overall effect of PUA on microzooplankton growth was negative, especially at the higher concentrations, but there were pronounced differences in response among common planktonic species. For example, the growth of *Codonella* sp., *Leegaardiella sol*, *Prorodon* sp., and *Gyrodinium spirale* was impaired at 2 nM, whereas *Strombidium conicum*, *Cyclotrichium gigas*, and *Gymnodinium* sp. were not affected even at 20 nM. These results indicate that PUA can induce changes in microzooplankton dynamics and species composition.

## 1. Introduction

Diatoms are dominant autotrophic plankton. They contribute 40% of the total ocean primary production and *ca.* 20% of global CO_2_ fixation [1,2,3]. Due to their relatively small surface to volume ratios, diatoms tend to dominate phytoplankton communities under nutrient-replete conditions, such as in coastal waters and upwelling zones [4], where they form the base of food webs and fisheries. Diatoms are responsible for significant vertical export of organic matter from the euphotic zone to the seafloor (e.g., [5]) and, therefore, are key players in the biogeochemical cycles of C, N, P, Si, and Fe [6].

Allelopathy (here production of chemicals by algae to inhibit the growth of competitors and deter grazers) is wide spread among phytoplankton ([7,8] and references therein) and has been repeatedly invoked as a crucial mechanism that leads to remarkable recruitment success in diatoms (e.g., [7]). Many diatom species produce a series of cytotoxic secondary metabolites collectively termed oxylipins, which result from decomposition of unsaturated fatty acids [9,10]. To date, fourteen oxylipin derivatives have been structurally identified in marine planktonic diatoms [11]. The polyunsaturated aldehydes (PUA) are the best studied group of these metabolites in terms of their effects on marine organisms. PUA were first identified in the diatoms *Skeletonema costatum*, *Pseudonitzschia delicatissima*, and *Thalassiosira rotula* [9] and subsequently in *Phaeocystis pouchetii* [12].

The centric diatom *Skeletonema marinoi* Sarno and Zingone, which has been recently separated from the *S. costatum* complex [13], produces several PUA including octadienal (OD) and heptadienal (HD) [14]. The enzymatic cascade leading to oxylipin production in *S. marinoi* is triggered when the integrity of cells is compromised [15]. It can produce PUA upon cell disruption during the exponential, stationary, and declining phase of its growth, with a maximum wound activated PUA production of up to 9.8 fmol·cell^−1^ [16,17]. During the final stages of an *S. marinoi* bloom, PUA production per cell was correlated with its abundance and cell lysis rates, suggesting a potential release of PUA into seawater without engaging the wound-activated cascade [18].

Most studies on diatom PUA cytoxicity have been dedicated to inhibitory effects on reproduction and development of marine invertebrates, such as copepods [9,19,20,21], sea urchins [22], and sea stars [23]. These studies reported deleterious PUA effects in experiments using monoclonal algal cultures. It was found in [24] that copepod reproduction was affected less when the animals were fed a mixed diet, which in addition to the cytotoxic *T. rotula* contained a non-toxic dinoflagellate.

Traditionally, mesozooplankton (*i.e.*, consumers between 200 and 2000 µm), such as planktonic copepods, have been considered the dominant grazers of diatoms [3]. Recent evidence shows, however, that microzooplankton herbivory is a major factor controlling primary production in the ocean [25]. Microzooplankton (*sensu stricto* consumers between 20 and 200 µm) mostly include protists such as ciliates and dinoflagellates that form biomass equal to that of mesozooplankton [26]. The ability of microzooplankton to feed on diatoms, including large and chain-forming forms, is well documented [27,28,29,30,31,32].

Flynn and Irigoien [33] have questioned the concept of “insidious” effect of diatom consumption upon copepods [9]. Using a modeling approach, they have demonstrated that killing the copepod offspring cannot be sustained as a defense mechanism since the probability of the copepod offspring consuming a cell of the same clone that induced hatching inhibition is lower than that of consuming a competitor or predator of that clone. Thus, PUA production would not confer any advantage to the diatoms if their main targets were copepod eggs. These authors have concluded that such a defense mechanism would make most sense against microzooplankton that feed and grow in time and spatial scales comparable to that of the diatoms. Thus, determining PUA effects on microzooplankton is key to understanding the ecological role of these chemicals in the ocean.

Plankton growth rate is a fundamental biological property and governs species composition, productivity, and carbon transformations in pelagic systems [34]. Knowledge of growth rates of individual species and their assemblages is critical to understanding food web responses to PUA. Therefore, the goal of this study was to determine the effects of dissolved diatom-linked PUA (OD and HD) on the growth of natural microzooplankton communities and their components in productive coastal waters. Specifically, this study sought to determine the effect of dissolved PUA on ciliates and dinoflagellates at the community and species-specific levels at the concentrations simulating the bloom of cytotoxic *S. marinoi*. To achieve these objectives, a set of PUA exposure experiments was conducted with natural plankton collected from the Chesapeake Bay and the Virginia coastal waters. Although the paucity of information on microzooplankton response to PUA made it difficult to formulate specific predictions, we expected that the overall effect of HD and OD on microzooplankton growth would be negative.

## 2. Results

### 2.1. Water Column Conditions and Ambient PUA Concentrations

The water column parameters and PUA concentrations at the study sites are presented in Table 1. The experimental samples collected in the Chesapeake Bay (Experiment, Exp. 1–3) were characterized by low salinity and temperature compared to the Atlantic coastal waters (Exp. 4 and 5). Dissolved HD varied from 0.004 nM in the Chesapeake Bay near Ragged Point to 0.06 nM in the Wachapreague Inlet. The Eastern Shore Lab (ESL) site had elevated particulate HD and OD. The latter parameter was undetectable in both the Choptank River and Ragged Point sites. The total PUA (*i.e.*, the sum of dissolved and particulate PUA) was also higher at the Atlantic coastal sites (0.077 and 0.080 nM in Exp. 4 and 5, respectively) than at the Chesapeake Bay sites (0.009 and 0.034 nM).

**Table 1:** The water column parameters and ambient PUA concentrations at the study sites. “nd” denotes no data.

Exp.	Date	Location		Temp. °C	Salinity	Heptadienal		Octadienal	
						Dissolved nM	Particulate nM	Dissolved nM	Particulate nM
1	Apr-13	Chesapeake Bay	Choptank River, HPL	14.0	10.0	nd	nd	nd	nd
2	May-14		Choptank River, HPL	16.7	9.4	0.021	0.002	0.011	0.000
3	May-14		Ragged Point	16.5	10.0	0.004	0.002	0.003	0.000
4	Sep-14	Atlantic waters	Wachapreague, ESL	23.2	29.8	0.014	0.055	0.004	0.004
5	Sep-14		Wachapreague, Inlet	24.0	30.0	0.060	0.017	0.001	0.002

### 2.2. Microplankton Composition and Biomass

**Figure 1:**
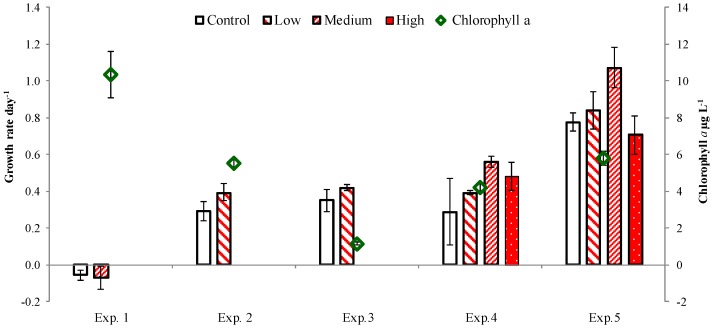
Initial chlorophyll *a* concentrations (diamonds, secondary axis) and dynamics (bars, primary axis) at different PUA concentrations in the experiments. Low = 2 nM and 0.2 nM, Medium = 5 nM and 0.5 nM, and High = 20 nM and 2 nM of heptadienal and octadienal, respectively, as described in Methods. The asterisk denotes significant difference from control.

**Figure 2:**
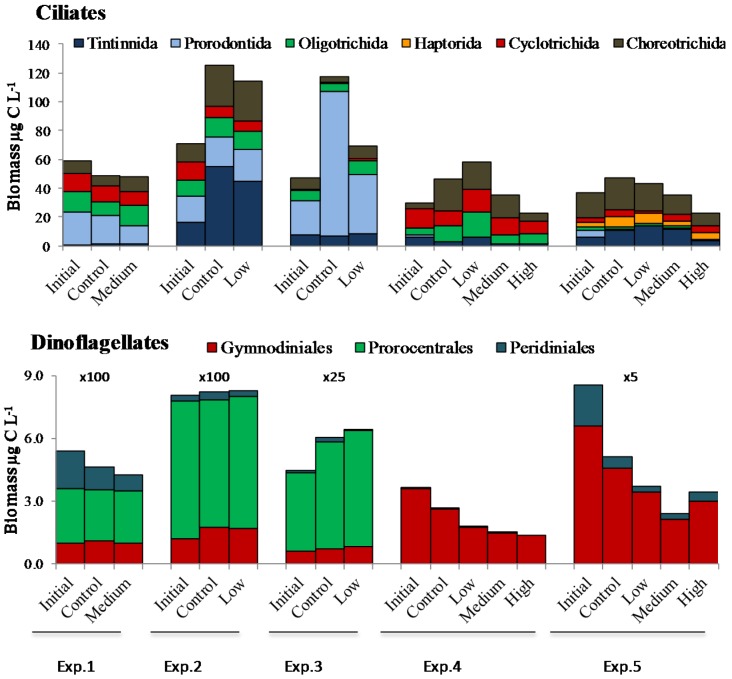
Microzooplankton biomass and taxonomic composition at the order level. Low, Medium, and High PUA concentrations correspond to Figure 1. Multipliers above the bars in the dinoflagellate panel refer to the *y*-axis.

Chlorophyll *a* initial concentrations (Figure 1) ranged from a maximum of 10.4 µg·L^−1^ in April 2013 (Exp. 1) to the minimum of 1.13 µg·L^−1^ in May 2014 (Exp. 3). In the Choptank River, diatoms (mainly *Rhizosolenia fragilissima* and *Pseudonitzschia cf. delicatissima*) and dinoflagellates (described below) were co-dominant, whereas at the Ragged Point site diatoms were replaced by cryptophytes (Exp. 3). In the Atlantic coastal waters (Exp. 4, 5, Figure 2) phytoplankton also were dominated by diatoms (mainly *Chaetoceros* spp.). In all experiments but one experiments (Exp. 1) chlorophyll *a* concentration increased during the experiments. However, it changed in response to PUA addition only in Exp. 5, where its increase rate was significantly higher in the medium treatment than in control (*p* < 0.01, Dunnett multiple comparisons test, Figure 1).

In Exp. 1 and 2 chloroplast-bearing prorocentroid and gymnodinoid dinoflagellates attained high abundance (~1 × 10^6^ cells·L^−1^) and biomass (Figure 2). The dominant species were *Prorocentrum minimum* and *Karlodinium veneficum* (formerly known as *K. micrum*), [35], which are known to produce high density blooms in the Chesapeake Bay. Overall, dinoflagellates contributed more than 90% of microzooplankton biomass in the Choptank River. Among ciliates, small and medium-sized species, such as the oligotrichs *Strombidium epidemum*, *S. acutum*, the prorodontid *Balanion comatum* and the tintinnid *Tintinnopsis nana* were dominant. The proportional composition of microzooplankton reflected a decrease in the contribution of prorodontids and peridiniides among ciliates and dinoflagellates, respectively, in PUA treatments compared to control (Figure 2). Microzooplankton biomass dynamics in the experiments are discussed in Section 2.3.

In Exp. 3, microzooplankton initial biomass was elevated (137 µg·C·L^−1^) despite the low chlorophyll *a* concentration. The dinoflagellates *P. minimum* and *K. veneficum* were dominant in terms of biomass, followed by the large prorodontid ciliate *Prorodon* sp. (*ca*. 100 µm in length, 18 µg·C·L^−1^), *T. nana* (6.4 µg·C·L^−1^), the choreotrich *Lohmanniella oviformis* (4.43 µg·C·L^−1^) and the oligotrich *Halteria* sp. (3.43 µg·C·L^−1^). In Exp. 2 and 3 the chloroplast-bearing cyclotrichid ciliate *M. rubrum* biomass exceeded >10 µg·C·L^−1^. A strong decline (compared to control) in the proportional biomass of *Prorodon* sp. was the main effect of PUA addition in Exp. 3. Among dinoflagellates, peridiniids declined also, whereas the proportion of *P. minimum* increased.

In the Atlantic coastal waters near ESL (Exp. 4), microzooplankton community was distinctly different from the Chesapeake Bay and dominated by ciliates, which formed *ca.* 90% of total microzooplankton biomass. In Exp. 4, *M. rubrum* was dominant (13.19 µg·C·L^−1^) with the large tintinnid *Favella panamensis* distant second (4.9 µg·C·L^−1^). Heterotrophic and mixotrophic oligotrichs were also abundant. In the high PUA treatment the proportions of oligotrichs (mainly the mixotrophs *Strombidium* sp. and *S. conicum*) and cyclotrichids (*M. rubrum*) peaked, whereas those of tintinnids and choreotrichids declined compared to control. Gymnodiniids formed more than 98% of dinoflagellate biomass; many of their cells were heterotrophic (aplastidic).

Microzooplankton community was more balanced in Exp. 5, where ciliates and dinoflagellates contributed to total biomass equally (43% and 57%, respectively). *Gymnodinium verruculosum* was the most abundant among dinoflagellates and despite its small size (15–20 µm) formed *ca.* 37% of dinoflagellates biomass. The heterotrophic choreotrich *Lohmanniella oviformis* was the most abundant among ciliates (10,615 cell·L^−1^), whereas the choreotrich *Strombidium neptuni* contributed disproportionally to total biomass (12 µg·C·L^−1^). The effect of PUA on the ciliate composition was the most pronounced in the high treatment, where haptorids (mainly the large mixotroph *Cyclotrichium gigas*) and *M. rubrum* increased proportionally at the expense of oligotrichs and tintinnids.

### 2.3. PUA and Community Growth Rates

**Figure 3:**
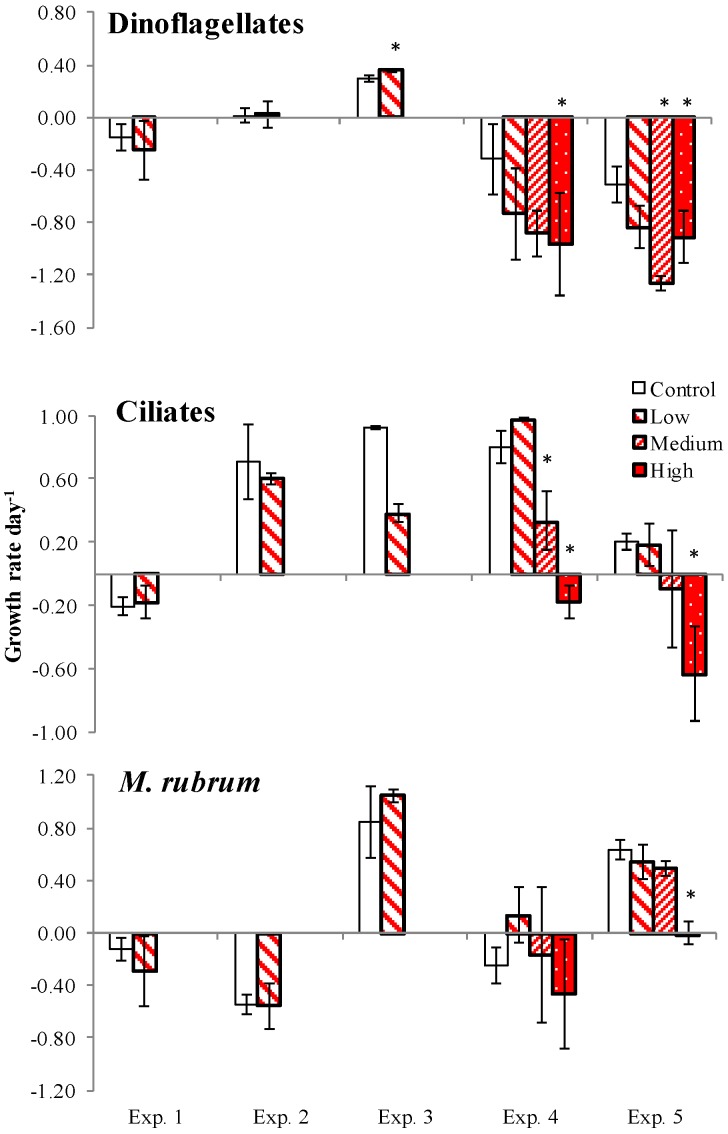
Growth rates based on biomass changes over time in PUA treatments and control for dinoflagellates, ciliates and *Mesodinium rubrum*. Asterisks denote the rates that were significantly different (*p* < 0.05) from control. Exp. 1–3: *t*-test, Exp. 4–5: Dunnett multiple comparisons test. The bar patterns denote Low, Medium, and High PUA concentrations as in Figure 1.

At the community level, ciliates grew in control in four out of five experiments (Figure 3), *M. rubrum* grew in Exp. 3 and 5, and dinoflagellates grew only in Exp. 3. Overall, microzooplankton tended to grow slower (or declined faster) in the PUA treatments than in control. However, in the Chesapeake Bay experiments, where PUA were added at low (Exp. 2 and 3) and medium concentrations (Exp. 1), their effect on dinoflagellates and ciliates was significant only in Exp. 3 (*p* < 0.05). In the three-concentration experiments in the coastal Atlantic waters, dinoflagellates declined significantly in both the medium and high PUA treatments in Exp. 5 and only in the high treatment in Exp. 4. Ciliates responded in reverse order (at medium and high concentrations in Exp. 4 and only at high concentration in Exp. 5). Considered separately, *M. rubrum* responded only to the high concentration of PUA in Exp. 5.

### 2.4. PUA and Species-Specific Growth Rates

**Figure 4:**
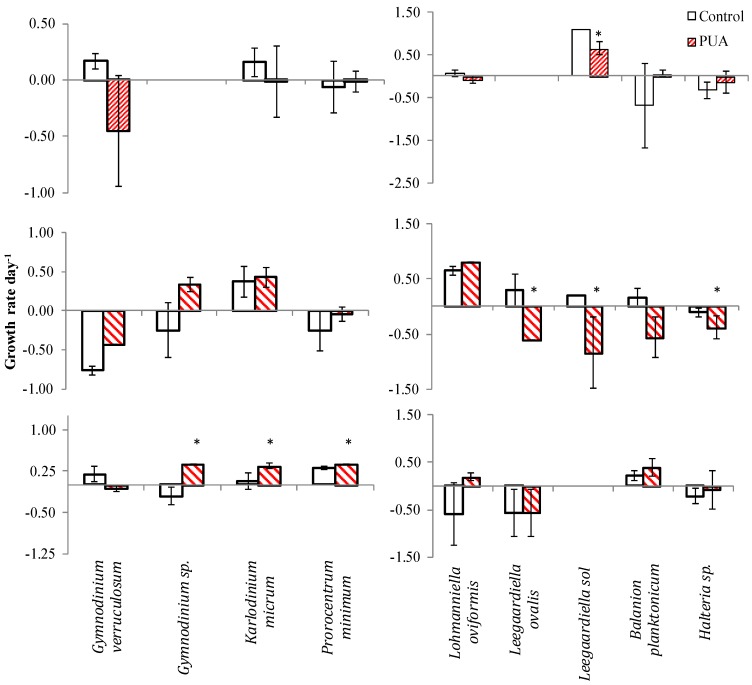
Growth rate in control and in PUA treatments for dinoflagellates (left panel) and ciliates species (right panel) during the three experiments conducted in the Chesapeake Bay area. Asterisks denote the rates that were significantly different from control (*t*-test <0.05). The bar patterns denote Low, Medium, and High PUA concentrations as in Figure 1.

Microzooplankton dynamics in response to PUA additions differed among the abundant species. The oligotrich congeners *Leegaardiella ovalis* and *L. sol* showed slower growth even at low PUA concentrations (Figure 4, Exp. 2). Other species (*P. minimum*, *Halteria* sp.) were unaffected at low and medium concentrations in the Chesapeake Bay experiments (Exp. 1–3) or had the opposite responses to the same concentration of PUA in Exp. 2 and 3 (*Gymnodinium verruculosum* and *Balanion planktonicum*). The dinoflagellate *K. veneficum* and the choreotrich ciliate *Lohmanniella oviformis* even appeared to be stimulated by low PUA additions in Exp. 3. The former species declined in the medium PUA treatment in Exp. 1.

The latter type of response was also observed for several species in Exp. 4 and 5 (Table 2).

**Table 2:** Microzooplankton species-specific growth rates (day^−1^) in control and PUA treatments in Exp. 4 and 5. The letters represent the result of Tukey’s pairwise comparison test. Shared letters denote no difference between the pairs (*p* < 0.05). PUA concentrations are as in Figure 1.

	Species	Control		Low		Medium		High	
**Exp. 4**	*Balanion planktonicum*	−1.06 ± 0.23	ab	−0.41 ± 0.15	a	−1.61 ± 0.10	b	−1.54 ± 0.15	b
	*Codonella* sp.	−1.33 ± 0.10	a	−2.82 ± 0.12	b	−2.83 ± 0.17	b	none	-
	*Cyrtostrombidium* sp.	1.43 ± 0.05	a	1.01 ± 0.07	a	−0.56 ± 0.52	b	−0.37 ± 0.12	b
	*Favella panamensis*	−0.75 ± 0.08	b	0.23 ± 0.11	a	−1.73 ± 0.13	c	−1.50 ± 0.16	c
	*Gymnodinium verruculosum*	−0.12 ± 0.48	a	−0.85 ± 0.47	b	−1.05 ± 0.42	b	−1.21 ± 0.47	b
	*Gymnodinium* sp.	−0.29 ± 0.05	a	−0.35 ± 0.05	a	−0.43 ± 0.20	a	−0.35 ± 0.22	a
	*Gyrodinium spirale*	0.65 ± 0.05	a	−0.33 ± 0.14	b	−1.35 ± 0.10	c	−1.62 ± 0.23	c
	*Lohmanniella oviformis*	0.62 ± 0.26	a	0.80 ± 0.23	a	0.44 ± 0.16	a	0.02 ± 0.11	a
	*Strobilidium neptuni*	2.50 ± 0.07	a	2.44 ± 0.17	a	2.08 ± 0.19	a	0.84 ± 0.25	b
	*Strombidium conicum*	0.83 ± 0.15	b	1.76 ± 0.17	a	0.90 ± 0.18	b	1.03 ± 0.06	b
	*Strombidium lynni*	1.62 ± 0.18	ab	2.14 ± 0.32	a	0.09 ± 0.14	bc	−0.43 ± 0.82	c
	*Strombidium* sp.	0.96 ± 0.05	a	1.11 ± 0.31	a	0.18 ± 0.20	a	0.69 ± 0.57	a
	*Strombidium acutum*	0.01 ± 0.15	ab	0.74 ± 0.18	a	−0.80 ± 0.45	bc	−1.35 ± 0.00	c
**Exp. 5**	*Cyclotrichium gigas*	0.46 ± 0.23	a	0.46 ± 0.46	a	0.35 ± 0.28	a	0.55 ± 0.45	a
	*Cyrtostrombidium* sp.	−0.47 ± 0.00	a	0.77 ± 0.12	a	−0.47 ± 0.00	a	−0.12 ± 0.28	a
	*Favella panamensis*	1.10 ± 0.00	a	1.10 ± 0.40	a	0.64 ± 0.23	a	none	-
	*Gymnodinium verruculosum*	−0.57 ± 0.07	a	−0.77 ± 0.09	ab	−1.19 ± 0.07	ab	−0.99 ± 0.16	b
	*Gyrodinium spirale*	−0.39 ± 0.09	a	−0.40 ± 0.08	a	−1.72 ± 0.07	c	−1.19 ± 0.10	b
	*Leegaardiella sol*	1.41 ± 0.04	a	0.85 ± 0.16	ab	0.84 ± 0.00	ab	0.59 ± 0.22	b
	*Lohmanniella oviformis*	0.19 ± 0.05	a	0.33 ± 0.12	a	−0.47 ± 0.05	b	−1.72 ± 0.21	c
	*Strobilidium neptuni*	−0.61 ± 0.12	a	0.08 ± 0.08	a	−0.82 ± 0.35	ab	−1.28 ± 0.17	b

**Figure 5:**
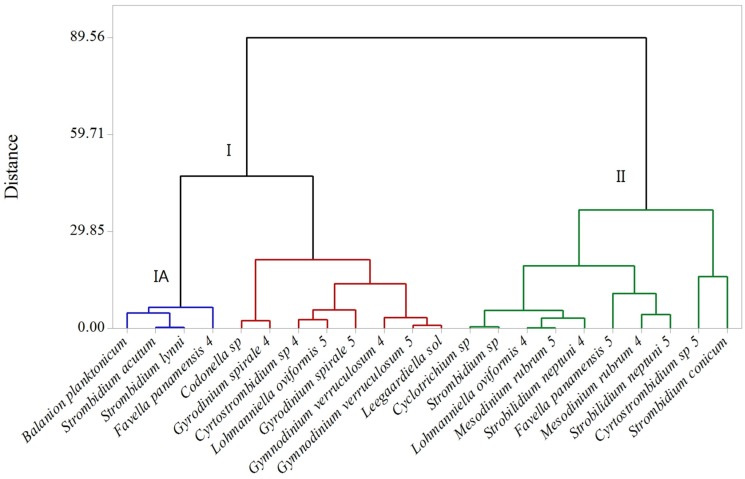
The results of cluster analysis of microzooplankton species-specific response to PUA in Exp. 4 and 5 expressed as the ratio of microzooplankton T_24_ abundance at each PUA concentration to that in control. Cluster **I** includes the species that responded negatively to PUA; Sub-cluster **IA** includes the species that increased in the low PUA treatment and declined in the medium and high treatments. Cluster **II** includes the species with a weak response, visible only at the high PUA concentration or no response at all. The numbers next to the species name refer to the experiment where these species occurred (see Table 2). Ward linkage and Pearson squared distance were used to build the cluster tree.

Based on their dynamics across the PUA concentration gradient in these experiments, microzooplankton were separated into two clusters (Figure 5). The first cluster (I) included species that demonstrated a distinctly negative reaction to PUA, including complete disappearance in the high PUA treatment for some (*Codonella* sp.). The growth rates of most microzooplankton in this cluster declined already at the medium concentration. The first cluster groups included four ciliates (*B. planktonicum*, *Strombidium acutum*, *S. lynni*, and *Favella panamensis* in Exp. 4, sub-cluster IA), which increased at low PUA and declined in the medium and high treatments. The second cluster (II) consisted of species that either did not respond to PUA addition at all (*Cyclotrichium gigas*, *Strombidium* sp., *S. conicum)*, or their response was weak and manifested only at the highest concentration (*Strobilidium neptuni*, *M. rubrum*). For most species that occurred in both experiments, their response to PUA was consistent. However, *L. oviformis*, *F. panamensis* and *Cyrtostrombidium* sp. reacted differently to the same PUA concentrations in Exp. 4 and 5.

**Figure 6:**
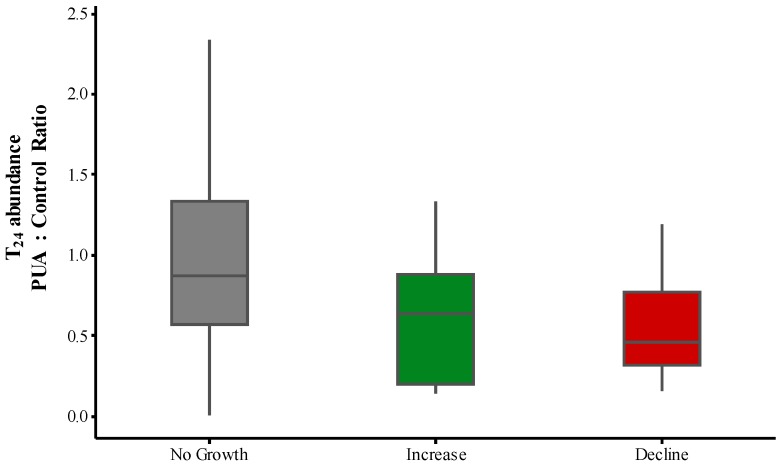
Microzooplankton species-specific response to the medium PUA concentration in Exp. 1, 4, and 5 expressed as the ratio of their abundance in PUA to that in control. The species were divided into three groups according to their dynamics in control: no change over 24 h, increase, and decline. Boxplots: the upper and lower whisker 25% of the distribution, interquartile range box: middle 50% of the data, line-median.

To test whether population growth dynamics had any effect on the species response to PUA we compared all 5 nM treatments with control. This single PUA level was selected because it was used in three experiments out of five. The ratio between abundance in control and the PUA treatment was similar between the groups of species that did not change in control over time, increased, or declined (Figure 6).

### 2.5. Methanol Addition Effect

**Figure 7:**
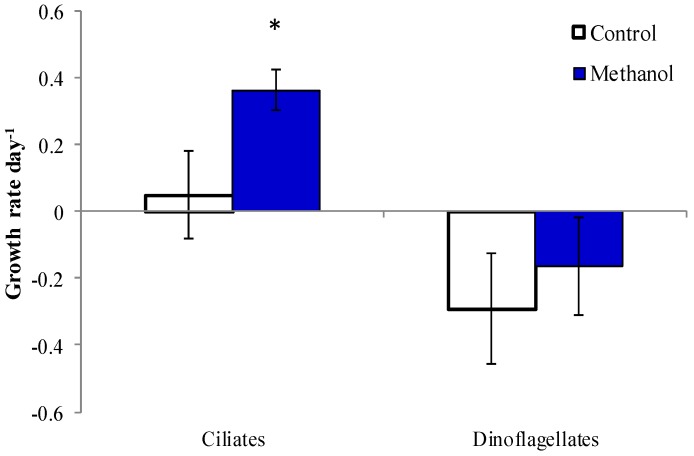
Microzooplankton growth rates in control and methanol treatment. The asterisk denotes significant difference compared to control (*t*-test <0.05).

The effect of methanol, which was used as a solvent for PUA, was tested on natural plankton from the Choptank River near HPL in October 2014 (Exp. 6). Two tintinnids, *Eutintinnus pectinus* and *Tintinnopsis beroidea*, the oligotrich ciliate *Tontonia gracillima*, and the athecate dinoflagellate, *Gymnodinium* sp., demonstrated higher growth rates in the methanol treatment compared to control (Table 3). Although no significant differences were observed for the rest of microzooplankton, the cumulative effect of minor changes resulted in the higher growth rate of ciliates at the community level (Figure 7, *p* < 0.05). The effect of methanol on dinoflagellates was not significant at the community level.

**Table 3:** Growth rates of microzooplankton in the methanol test. *p*-Values are from *t*-test of control *vs.* methanol.

Species	Control	Methanol	*p*-Value
*Akashiwo sanguinea*	−0.11 ± 0.15	−0.15 ± 0.11	ns
*Balanion comatum*	−0.19 ± 0.13	0.18 ±0.06	0.06
*Balanion planktonicum*	0.17 ± 0.23	0.56 ± 0.08	ns
*Cyclotrichium gigas*	0.22 ± 0.11	−0.38 ± 0.28	ns
*Eutintinnus pectinis*	−0.48 ± 0.37	0.70 ± 0.25	0.05
*Gymnodinium verruculosum*	−0.62 ± 0.04	−0.45 ± 0.07	ns
*Gymnodinium* sp.	−0.93 ± 0.17	0.10 ± 0.06	0.00
*Gyrodinium dominans*	−1.15 ± 0.23	−0.65 ± 0.14	ns
*Gyrodinium uncatenatum*	−0.84 ± 0.41	0.10 ± 0.04	ns
*Leegaardiella sol*	0.60 ± 0.24	1.12 ± 0.07	ns
*Lohmanniella oviformis*	0.72 ± 0.22	1.29 ± 0.12	ns
*Mesodinium acarus*	−0.50 ± 0.14	−0.41 ± 0.20	ns
*Polykrikos schwartzii*	0.16 ± 0.09	−0.21 ± 0.32	ns
*Strombidium acutum*	0.43 ± 0.12	0.69 ± 0.07	ns
*Strombidium conicum*	−0.09 ± 0.16	−0.91 ±0.42	ns
*Strombidium* sp.	−0.74 ± 0.45	−0.80 ± 0.50	ns
*Tintinnopsis beroidea*	−0.14 ± 0.07	0.81 ± 0.00	<0.01
*Tintinnopsis campanula*	−0.79 ± 0.32	−0.12 ± 0.17	ns
*Tintinnopsis coronata*	1.23 ± 0.42	1.83 ± 0.37	ns
*Tintinnopsis denticulata*	−1.90 ± 0.00	−0.97 ± 0.46	ns
*Tintinnopsis minuta*	−0.54 ± 0.33	0.08 ± 0.07	ns
*Tontonia gracillima*	−0.16 ± 0.09	0.31 ± 0.12	0.03

“ns” denote no significant difference between treatments.

## 3. Discussion

The growth rates of ciliates and dinoflagellates observed in control samples were within the range reported for natural microzooplankton populations in temperate and polar waters ([36] and references therein). Due to their competitive and/or predator-prey interactions and different resource requirements, multiple populations comprising microzooplankton may oscillate out of phase, whereas short-term incubations provide only a snapshot of these dynamics. Therefore, it is not surprising that only part of the microzooplankton community increased over the 24 h period in this study. The importance of a species-specific approach cannot be overemphasized in field allelopathy effect experiments. Counting microzooplankton into size classes or broad taxonomic categories (e.g., ciliates and dinoflagellates) could have masked many of their responses.

It is apparent that methanol was not responsible for the inhibitory effects of PUA on microzooplankton growth. In fact, it could have alleviated their impact to a certain degree. Methanol concentrations in this study were well below the threshold of 7 µL·mL^−1^, above which diatom growth can be inhibited [14]. At concentrations below 1% methanol had no inhibitory effect on the growth of ruminal bacterial strains [37]. It is not clear why 0.1% (final concentration) methanol addition stimulated the growth of several ciliate species in our test experiment. It should be mentioned that methanol is a significant volatile organic molecule involved in the biogeochemical cycle of carbon in the ocean via methylotrophic bacteria [38]. Further, recent research indicates that several cyanobacteria and eukaryotic phytoplankton species can produce pulses of methanol up to 3 fmol·cell^−1^ [39]. Thus, it is not impossible that methanol addition stimulated the growth of some prey microorganisms that in turn stimulated the ciliates. On the other hand, [40] found that methanol increased PUA production in their diatom culture.

To our knowledge, this study is the first attempt to experimentally examine the species-specific growth responses of natural microzooplankton to diatom-linked OD and HD. In laboratory culture experiments with PUA-producing strains of *S. costatum* and *T. rotula* and herbivorous protists, the survival and growth of two dinoflagellate grazers were not consistently related to growth stage or PUA production potential [41]. However, dissolved substances released by the diatoms in these experiments had negative effects on the ciliates *Strombidium acuminatum* and *Favella ehrenbergii*, which preferably preyed on non-PUA producing strains of the above species. Microzooplankton growth rates at a given temperature were lower when colonial *P. pouchetii* and/or *T. rotula* were abundant in the Barents Sea [42]. It should be noted that both phytoplankton species also are capable of producing decadiental, a potent PUA cytotoxin [9,43] not produced by *S. marinoi*.

The highest concentrations of dissolved HD and OD in natural samples were measured during the *S. marinoi* bloom in the Adriatic Sea (18.13 nM and 10.11 nM, respectively) [44]. Much lower background concentrations were measured in the Strait of Gibraltar [45] and in the offshore Atlantic Ocean waters [46] where no bloom was recorded. In the latter two cases, PUA production by cells >10 µm was in the pico-molar range. Our results (0.01–0.08 nM) are within the range of background concentrations reported for productive coastal waters. This study simulated a natural range of PUA concentrations that plankton can be exposed to in productive coastal waters during a bloom of cytotoxic of *S. marinoi*. The low, medium, and high concentrations correspond to the light, moderate, and heavy blooms of PUA-producing diatoms (2.7, 6.7, and 27 × 10^6^ cells L^−1^, respectively) based on PUA production of 7.5 fmol cell^−1^ by the cytotoxic strain of *S. marinoi* [16] and the assumption that 10% of cells undergo lysis and release PUA [47]. In the natural waters, PUA-producing diatoms can reach the abundance of 10^7^ cells L^−1^ [18,48]. Therefore, the PUA concentrations used in this study are ecologically realistic [47]. It should be noted that several published PUA exposure studies used much higher concentrations between 3.2 µM and 145 µM [43,48,49,50,51]. The OD:HD ratios up to 1:1 were reported in laboratory experiments [16]. However, [52] observed the OD:HD ratio of 1:30 in a mesocosm study. A shift towards HD production can occur under natural conditions [53]. The ratio of 1:10 used in our experiments appears to be a reasonable compromise between the values reported from the cultures and field observations.

Although the ambient PUA concentrations measured in this study confirm that no cytotoxic bloom related to HD and OD was in progress at the time of experiments, they also may indicate that such conditions could have occurred at an earlier date. Specifically, the total concentration of PUA at the Atlantic coastal sites (Exp. 4 and 5) correspond well to the residual level of 0.1 nM that can persist in the water column following a cytotoxic bloom event [44]. Part of the microzooplankton communities we examined demonstrated resilience to the diatom-linked cytotoxins. The question then arises: how do PUA-exposed and PUA-naïve microzooplankton respond to the same concentrations of PUA? In areas frequently exposed to cytotoxic diatom blooms, microzooplankton may have adaptations to avoid cytotoxic cells and detoxify PUA. Although there is no published information on PUA in the Chesapeake Bay and the Virginia coastal waters apart from this study, *Skeletonema* is common in both ecosystems and occasionally forms blooms.

The mechanism of PUA effects on microzooplankton growth rates remains unclear. PUA can act as potent mitotic inhibitors and pro-apoptotic agents [23]. At the typical bloom concentrations, PUA can inhibit the growth of diatoms [48] and other phytoplankton [14]. Since phytoplankton are the main food source for microzooplankton, their decline could negatively affect the growth of ciliates and dinoflagellates. However, the dynamics of chlorophyll *a* in our experiments do not support the assumption that food limitation caused microzooplankton growth rate decline in the PUA treatments. Although total chlorophyll *a* may be too crude a measure to describe the specific resource requirements of individual microzooplankton species, its dynamics correlated with microzooplankton biomass dynamics in this study. Interestingly, this correlation had the opposite signs in the Chesapeake Bay and the Atlantic coast experiments. In the former set of experiments, the growth rates of both ciliates and dinoflagellates positively correlated with the chlorophyll-based rates (Pearson *r* = 0.86 and 0.85, respectively, *p* < 0.05). It should be noted that the dominant dinoflagellates in the Chesapeake Bay were plastidic. Hence they were contributing to total chlorophyll *a*, particularly when diatom abundance was low (Exp. 3). In the Atlantic coast experiments (Exp. 4 and 5), the growth rates of ciliates and the predominantly heterotrophic dinoflagellates displayed a weak negative relationship with chlorophyll *a* dynamics (*r* = 0.57 and 0.62, respectively, *p* > 0.05).

The complexity of food web interactions presents an obvious challenge in field PUA experiments with natural plankton. Although potential mesozooplankton predators were removed from the experimental containers, some metazoan zooplankton, such as rotifer and copepod nauplii, remained and their presence could have affected directly or indirectly microzooplankton growth. However, their numbers were too low to yield reliable abundance estimates using the microzooplankton methods and sample volumes. Some PUA such as decadienal can inhibit the flagellar activity of starfish sperm cells [54]. Although no data exists on PUA effects on microzooplankton swimming activities, a similar effect could expose the affected microzooplankton to increased predation risk. On the other hand, behavioral changes in the invertebrate feeding activities could have released microzooplankton from top-down control. This could potentially explain the stimulating effect of low PUA concentrations on some ciliates and dinoflagellates in this study. Incubation experiments with natural plankton often yield low net growth estimates for microzooplankton due to intraguild predation within their communities (e.g., [55,56,57]). For example, the dominant dinoflagellates in this study, *P. minimum and K. veneficum*, are also prey for other microzooplankton [58]. The same applies to *Balanion* spp., *Lohmanniella oviformis*, and other small-sized ciliates, which can be kept below their reproductive potential by larger microzooplankton in bottle experiments [25,36,56,59]. In this study, some nanoplankton -sized ciliates, e.g., *Lohmanniella oviformis*, grew faster at low and intermediate PUA concentrations than in control bottles, indicating the presence of trophic cascades. For example, a positive response of these ciliates and the dominant dinoflagellates to low PUA addition in Exp. 3 coincided with a decrease in the growth of the large raptorial ciliate *Prorodon* sp. in the PUA treatment.

Further, it remains to be seen whether direct encounters with cytotoxic diatoms produce a similar effect on microzooplankton as dissolved PUA. In this study PUA was dissolved in a relatively large volume (compared to the average microplankton cell size), but bulk volumetric concentrations of a specified toxin may or may not be relevant when considering their potential effect on adjacent trophic levels [60]. Local concentrations in the immediate surroundings of PUA-producing diatom cells could be higher due to a low diffusion process away from the producer [14,44]. For example, *S. marinoi* growth was not affected by added dissolved domoic acid (DA), but inhibited in a mixed culture with a DA-producing strain of *Pseudo-nitzschia* [61]. To determine the impact of PUA-producing diatoms on organisms directly feeding on or surrounding them one would have to take into account microscale interactions among plankton.

A combination of field and laboratory experiments may be needed to unravel the complexity of PUA-microplankton relationship. Cultures may select for clones that are acclimated to grow on specific food sources under laboratory conditions, which may not be optimum, and, therefore, fail to elicit a maximum growth response in microzooplankton in contrast to their field populations (e.g., [36,62,63]). Further, *in situ* experiments might yield different inferences of underlying PUA relationships for natural populations than data derived from laboratory experiments with individual cultures [64]. However, culture studies offer indisputable advantages, such as the ability to control growth conditions, isolate specific factors, and apply methodologies that are not readily available or suitable for field conditions. Model organisms for culture studies should be chosen carefully taking into account the observed species-specific difference in microzooplankton response to PUA. Although pelagic oligotrichs can be difficult to maintain in cultures (e.g., [65]) they should be included in such experiments along with other critical components of microzooplankton, such as the mixotrophic and heterotrophic dinoflagellate taxa examined in this study.

## 4. Experimental Section

### 4.1. Field Sampling

**Figure 8:**
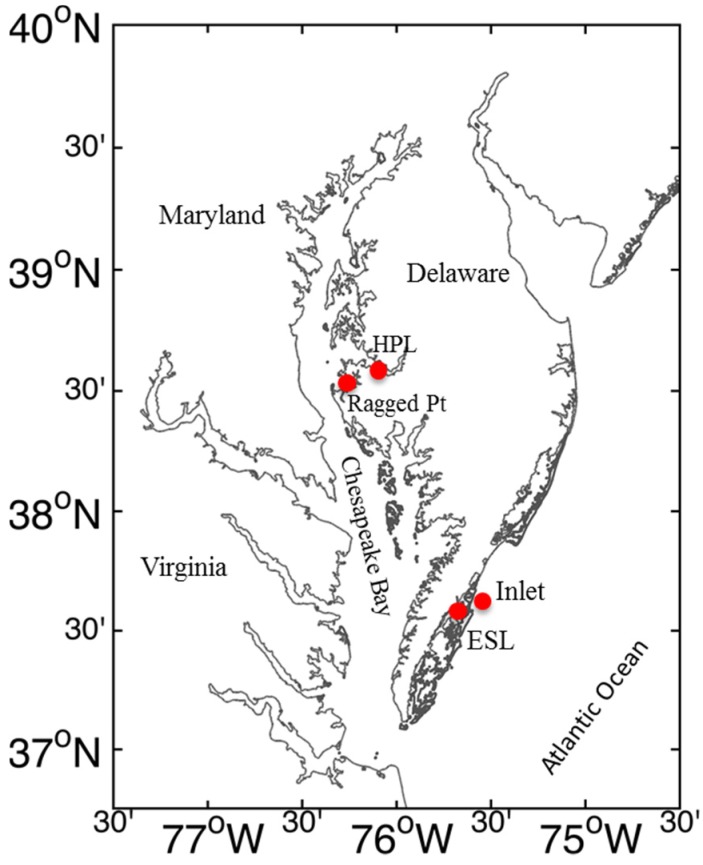
The study sites in the Chesapeake Bay and the Atlantic coast. HPL = Horn Point Lab; Inlet = Wachapreague Inlet; ESL = Eastern Shore Lab.

Seawater for experiments was collected from the Choptank River, a tributary of the Chesapeake Bay, from the dock at the Horn Point Laboratory of the University of Maryland Center for Environmental Science (HPL, Cambridge, MD, USA, Figure 8) in April 2013. In May 2014, seawater was also collected near the Choptank River and additionally from the Chesapeake Bay at Ragged Point. In September 2014, seawater was collected from two Atlantic Ocean coastal locations: one near the Virginia Institute of Marine Science Eastern Shore Laboratory (ESL, Wachapreague, VA, USA) and the other in Wachapreague Inlet. Prior to sampling, all glass-ware, plastic containers, and tubing were soaked in 10% HCl and rinsed with copious amounts of deionized water and then seawater. Gloves were used whenever handling experimental containers. At each station, water temperature and salinity were measured using hand-held YSI probes (Model 30 and Pro2030DO). Surface seawater was collected with a plastic bucket and carefully syphoned into 20 L polycarbonate carboys using submerged silicone tubing. During April 2013 and May 2014 experiments, the carboys were immediately transported to a temperature-controlled cold room at HPL, which was adjusted to ambient (±1 °C) water temperature. In September 2014, the water temperature was nearly equal to room temperature at ESL.

### 4.2. Experimental Setup

All manipulations were conducted under dim light, and samples were stored in a closed cooler whenever not being handled. The collected water was carefully screened through a 200-µm mesh net to remove larger zooplankton such as copepods. Post-incubation screening indicated that this technique was effective in removing mesozooplankton albeit rotifers, copepod nauplii, and some invertebrate larvae were present in some of the bottles. Triplicates of control and PUA treatments were prepared by carefully adding the experimental water to 0.61 L Nalgene clear polycarbonate bottles.

All samples were amended with L1 growth media diluted 1:1000 (final concentration). The bottles were closed with caps lined with plastic wrap to prevent air headspace and screened with neutral density filters to mimic ca. 25% surface irradiance. In Exp. 1–3, the bottles were mounted on a plankton wheel (~0.25 rpm) and incubated for 24 h under ambient temperature and light conditions in a walk-in incubator. In Exp. 4 and 5, the bottles were screened with a neutral density filters to mimic ~25% of incident radiation and incubated in oyster floats in seawater at the ESL dock. 

A mixture of two PUA, *trans*,*trans-*2,4-octadienal (OD, W372102, Sigma-Aldrich, Saint Louis, MO, USA) and *trans*,*trans*-2,4-heptadienal (HD, 180548, Sigma-Aldrich, Saint Louis, MO, USA) was added to the experimental bottles at the OD:HD ratio of 1:10. In Exp. 1, experimental samples were inoculated with 5 nM of HD and 0.5 nM of OD (final concentration). In Exp. 2 and 3, the concentrations of HD and OD were 2.0 and 0.2 nM, respectively. In Exp. 4 and 5, three different concentrations were used: 2, 5, and 20 nM of HD and 0.2, 0.5, and 2 nM of OD. Throughout the text, the above concentrations are designated as “low”, “medium”, and “high”, respectively. To prepare experimental inoculates of PUA, commercial stocks of HD and OD were diluted with anhydrous methanol (Sigma-Aldrich, Saint Louis, MO, USA). The final concentrations of methanol in seawater corresponding to the low, medium, and high PUA concentrations were 0.10, 0.21 and 1.00 µL·mL^−1^. To test the effect of methanol on planktonic protists, we added methanol at 1 µL·mL^−1^ (final concentration) to triplicated bottles with natural microzooplankton collected from the Choptank River (near HPL) in October 2014 (Exp. 6) and incubated them along with untreated controls for 24 h as described above.

### 4.3. Chlorophyll A Analysis

For chlorophyll *a* analysis, 50–150 mL of seawater was filtered onto 0.7 µm 25 mm Whatman GF/F filters. Chlorophyll *a* was extracted in 90% acetone for 24 h at −20 °C and measured using the acidic method [66] on a Turner Designs 10AU fluorometer (except Exp. 1, where the non-acidic method [67] and a Turner Designs TD-700 fluorometer were used).

### 4.4. Microzooplankton Analysis

Microzooplankton samples were collected at the beginning and end of experiments, preserved in 2% (final concentration) acid Lugol’s iodine, stored at 4 °C in opaque containers, and post-fixed with 1% (final concentration) formaldehyde after 24 h. Microzooplankton were settled onto Utermöhl chambers and counted under an Olympus IX-70 inverted microscope equipped with differential interference contrast (DIC), epifluorescence, and a digital camera. The entire surface area of a chamber was scanned at 200×. Protists were identified tentatively to the lowest possible taxonomic level consulting [68,69,70,71,72]. At least 40 individual cells within each abundant taxon were sized with an eyepiece micrometer at 400–600×. All ciliates were included in the counts, whereas dinoflagellates <15 µm in maximum dimension were not [73]. The smallest abundant ciliates in this study were *ca.* 15 µm, whereas dinoflagellates extended into the nanoplankton range. Recent literature indicates that most plastidic dinoflagellate genera found in this study are capable of phagotrophy [28,63,74,75]. Therefore, they were included in microzooplankton. Microzooplankton biovolumes were calculated from their linear dimensions by approximating geometric shapes [76] and converted to carbon [77]. Tintinnid volumes were calculated based on their cell dimensions; empty loricas were disregarded.

### 4.5. Rate Calculations

Microzooplankton instantaneous population growth rates (µ, day^−1^) were determined from the initial (*n*_0_) and final (*n*_t_) abundances of each morpho-species and incubation time (*t*, day) assuming exponential growth:

µ = ln (*n*_t_/*n*_0_)/*t* (1)

Total ciliate and dinoflagellate community growth rates were calculated similarly based on their combined biomass. The ratio between the *T*_24_ abundance of different ciliate and dinoflagellate species in control and PUA treatments was calculated as a measure of their response to aldehyde addition (Figure 5 and Figure 6). Since experimental treatments and controls share the *T*_0_ abundance, this ratio can be treated as a proxy of growth. Its advantage for some statistical analyses is the absence of negative values, which are common for calculated growth rate when a population declines. Phytoplankton growth rates were calculated based on the changes in chlorophyll *a* concentrations over time using Equation (1).

### 4.6. Ambient PUA Analyses

The determination of dissolved and particulate PUA was performed based on a modification [40] of the Wichard protocol [17]. Volatile PUA were derivatized with *O*-(2,3,4,5,6-pentafluorobenzyl) hydroxylamine hydrochloride (PFBHA·HCl). To determine particulate production, 0.4 to 1.2 L of seawater was filtered onto GF/C filters. The harvested phytoplankton cells were re-suspended in 1 mL of a 25 mM PFBHA solution in Tris-HCl 100 mM (pH 7.2) containing 5 μL of internal standard (benzaldehyde, 0.1 mM in methanol). The cells were disrupted by 1 min of pulsed sonication (0.5 s ultrasound–0.5 s break cycle) to initiate PUA production. After incubation for 4 h at room temperature the samples were stored at −20 °C. Dissolved PUA was extracted from 0.4 to 1.2 L of seawater on PFBHA preloaded EASY^®^ solid phase extraction cartridges (Macherey-Nagel, Düren, Germany). After elution with PFBHA in methanol (5 mM), the samples were incubated for 1 h at room temperature to ensure complete derivatization and stored at −20 °C. The extraction phase was performed in the laboratory and the samples were then stored at −80 °C until analysis via gas chromatography-mass spectrometry (GC-MS).

Derivatized PUA were analyzed using a Thermo Trace 1310 gas chromatograph coupled to an ISQ single quadrupole mass spectrometer. The aldehydes were separated using a TG-5MS capillary (30 m × 0.25 mm × 0.50 µm) column from Thermo Scientific at a constant flow rate of 1.4 mL·min^−1^. Helium was used as the carrier gas. The GC conditions were as follow: initial temperature was set to 60 °C and hold for 2 min. The oven temperature was then raised to 240 °C at 8 °C min^−1^. A second ramp was applied at a rate of 15 °C·min^−1^ to reach a final temperature of 280 °C which was held for 2 min. The injection temperature was fixed at 250 °C and the injection mode was set to splitless. For the MS analysis, the mass spectra were acquired using electron impact (EI) ionization in positive ion mode. The ion source and the interface temperatures were respectively set to 200 °C and 250 °C. Authentic derivatized PUA standards and benzaldehyde (internal standard) were run to identify their retention time as well as their fragments by defining a scan range from 40 to 500 amu. Then, a single ion monitoring (SIM) was performed by monitoring: (i) molecular ions 301, 305, and 319 for benzaldehyde, heptadienal, and octadienal respectively; and (ii) fragments 271 and 276, respectively, for benzaldehyde and heptadienal/octadienal. GC-MS data were acquired and processed using Xcalibur software. Derivatized PUA were identified using the NIST 11 library.

### 4.7. Statistical Analyses

Rare taxa (here, less than 50 cells L^−1^ in the initial sample) were excluded from growth rate calculations to avoid statistically unreliable rate estimates. In several cases we settled additional samples to increase count reliability. Standard deviation is used as a measure of dispersion throughout the study. Experimental and control data were analyzed via Student’s *t*-test in experiments with a single PUA treatment. The effects of multiple concentrations were analyzed using one-way analysis of variance (ANOVA) and the means were separated using Tukey and Dunnett pairwise comparison tests. The response of microzooplankton to PUA at the species-specific level was examined using cluster analysis and boxplots. The relationships between microzooplankton growth and chlorophyll *a* were analyzed using Pearson product-moment correlation. All statistical analyses were performed using Minitab 17.

## 5. Conclusions

The results of the present study indicate that PUA can induce complex changes in microzooplankton growth dynamics and community composition that go beyond growth inhibition. The compositional changes may, in turn, alter food web structure and trophic interactions with positive feedbacks that might exacerbate PUA-producing phytoplankton blooms. Thus, the effects of diatom-produced PUA on microbial food web processes can be significant and thus warrant further detailed investigation.
